# Human iPS cell-engineered three-dimensional cardiac tissues perfused by capillary networks between host and graft

**DOI:** 10.1186/s41232-018-0084-7

**Published:** 2018-10-10

**Authors:** Hidetoshi Masumoto, Jun K. Yamashita

**Affiliations:** 1 Clinical Translational Research Program, RIKEN Center for Biosystems Dynamics Research, 2-2-3 Minatojima-minamimachi, Chuo-ku, Kobe, Hyogo 650-0047 Japan; 2 Clinical Translational Research Program, RIKEN Center for Developmental Biology, Kobe, Japan; 3 Department of Cell Growth and Differentiation, Center for iPS Cell Research and Application (CiRA), Kyoto University, 53 Shogoin Kawahara-cho, Sakyo-ku, Kyoto, 606-8507 Japan; 4 Department of Cardiovascular Surgery, Kyoto University Graduate School of Medicine, Kyoto, Japan

**Keywords:** Capillary network, 3D cardiac tissues, Human iPS cells, Cardiac regeneration

## Abstract

Stem cell-based cardiac regenerative therapy is expected to be a promising strategy for the treatment of severe heart diseases. Pluripotent stem cells enabled us to reconstruct regenerated myocardium in injured hearts as an engineered tissue aiming for cardiac regeneration. To establish a long-term survival of transplanted three-dimensional (3D) engineered heart tissues in vivo, it is indispensable to induce microcapillaries into the engineered tissues after transplantation. Using temperature-responsive culture surface, we have developed pluripotent stem cell-derived cardiac tissue sheets including multiple cardiac cell lineages. The application of gelatin hydrogel microsphere between the cell sheet stacks enabled us to generate thick stacked cell sheets with functional vascular network in vivo. Another technology to generate 3D engineered cardiac tissues using cardiac cells and biomaterials also validated successful induction of vascular network originated from both host and graft-derived vascular cells.

## Background

Stem cell-based cardiac regeneration is a rapidly expanding paradigm to deliver therapeutic approaches for severe cardiac disorders resistant to current therapies [1, 2]. The discovery of human induced pluripotent stem cells (iPSCs) [3] opened the door toward in vitro formulation of human myocardium aiming for cardiac regeneration. Engineered three-dimensional (3D) myocardial tissue constructs generated from human iPSCs including multiple cardiovascular lineage constructs are more likely to replicate the dynamic organization and function of native myocardium and have emerged as a robust methodology to accomplish myocardial regeneration in animal heart disease models [4–7]. In the context of cell transplantation to the heart, the 3D construct is reported to be advantageous over single cell injection into the myocardium because of the avoidance of mechanical loss related to the cell injection [8] and/or the higher survival efficiency *in vivo* [9].

In addition with the biophysical advantages of 3D structure in cell retainment after transplantation as described above, the introduction of microcapillaries is an important factor for long-term survival of the transplanted tissue. It is assumed that the transplanted tissue survives only through the direct diffusion of oxygen and nutrition at the initial stage of the transplantation, and vascular formation perfusing the whole engineered tissue would be indispensable for the long-term survival. It means that the successful cardiac regenerative strategy requires re-vascularization mechanisms to validate long-term myocardial regeneration.

### Cell sheet-based thick cardiac tissues

Cell sheet formulation is one of the principal methods to generate 3D tissues from single cells [10]. Okano et al. reported a novel method to generate cell sheets using poly (N-isopropylacrylamide) (PIPAAm), a temperature-responsive polymer which changes the property of culture surface from hydrophobic to hydrophilic along with the lowering of the temperature which enables us to collect the confluent cell culture as a cell-sheet shape preserving attachment molecule and extracellular proteins without enzymatical digestion or physical damage [11]. Using this method, we have reported a formulation of human iPS cell-derived “cardiac tissue sheet (CTS)” including multiple cardiac cell lineages including cardiomyocytes and vascular cells (vascular endothelial cells, and mural cells), and a successful human myocardial regeneration and functional recovery mainly mediated by paracrine mechanisms such as angiogenesis in a rat myocardial infarction model [6]. However, the extent of the engraftment was not fully satisfactory requiring additional strategies to enhance the regenerative capacity.

**Fig. 1:**
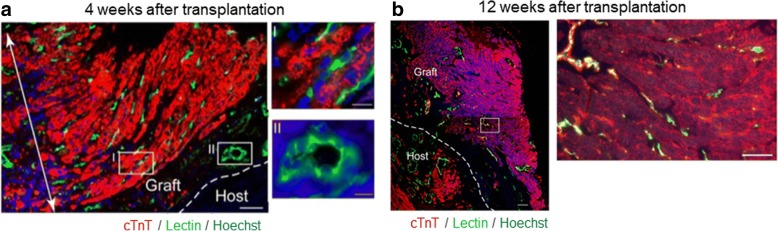
Vascular network formation after mouse embryonic stem cell-derived thick cardiac tissue sheets with gelatin hydrogel microsphere. cTnT immunostaining for lectin (represents perfused vasculature)-perfused rat heart. Cardiomyocytes (cTnT, red), perfused vessels (lectin-stained, green), and pre-stained graft nuclei (Hoechst, blue). **a** Four weeks after transplantation. Note that thick regenerated myocardium (double-headed arrow) supported by dense perfused capillary networks (green) were formed. Capillaries (I) and larger vessels (II) in the graft (green) were largely Hoechst-positive. **b** Twelve weeks after transplantation. High magnification image of white box in the right panel. A compact myocardial tissue with capillary vessels was formed. cTnT, cardiac troponin T. Scale bars 100 μm in (**b**) (left), 50 μm in (**a**) and (**b**) (right), 10 μm in (**a**) (I-II). Referred from reference no. 13 with modifications

It is also reported that the tissue thickening by the simple layering of cell sheets is limited for less than four layers because of the central necrosis due to the shortage of oxygen and nutrition supply [12]. To overcome this problem, we developed a cell-sheet stacking method to insert gelatin hydrogel microsphere (GHM), which is a biomaterial to work as a spacer between the cell sheets securing oxygen and nutrition supply among the whole cell sheet stacks. Using this method, we have reported a successful myocardial regeneration using mouse embryonic stem cell-derived CTSs in a rat MI model [13]. The engraftment efficiency of the stacked cell sheets with GHM was > 10 times higher than those without GHM. We also revealed that the transplantation of GHM-supported CTS stacks was secured by microcapillary network between host and graft which was verified by the injection of fluorescent dye-conjugated lectin via venous system of the host which stained the capillaries inside the graft at 4 weeks after transplantation (Fig. 1a). We stained the nuclei of cells consisting CTSs with Hoechst 33342 before transplantation and confirmed that the vasculature perfusing the engrafted tissues were composed of graft-originated vascular cells. At 3 months after CTS-stack transplantation, the graft seems to be similar with bona fide myocardium perfused with vasculature allocated in every 50 μm which is supposed to be sufficient for the perfusion of the whole regenerated myocardium (Fig. 1b).

Another method to induce vascular networks into the cell sheet-based 3D structures is to generate “vascularized cardiac cell sheets” using bioreactors [14, 15]. In the study, the authors developed a bioreactor system using a femoral muscle-based and a synthetic collagen gel-based vascular beds which can provide fair perfusion throughout the 12-layered cell sheets. This novel system may also serve as a technology to induce vascular networks inside the engineered tissues.

### Biomaterial-supported engineered cardiac tissue

Another format of 3D cardia tissue is biomaterials-supported engineered tissues which strongly support the structural stiffness of the artificial tissue structure [4, 16–18]. Taking advantages of a 3D cardiac tissue formation technology using rat [19] or chick [20] embryonic cardiac cells and a combination of biomaterials (collagen I, Matrigel), we have developed self-pulsating human iPS-derived engineered cardiac tissues (hiPSC-ECTs) with cylindrical [5] and mesh-like [7] shapes. The incorporation of multiple cardiac cell lineages into the hiPSC-ECTs enhanced the tissue function including tissue stiffness evaluated by the measurement of Young’s modulus, cardiomyocyte alignment, and sarcomeric ultrastructural maturation shown by transmission electron microscopy. The formulation of multiple lineages also validated a better force-frequency relationship which is known to be a parameter for tissue maturation [21].

**Fig. 2:**
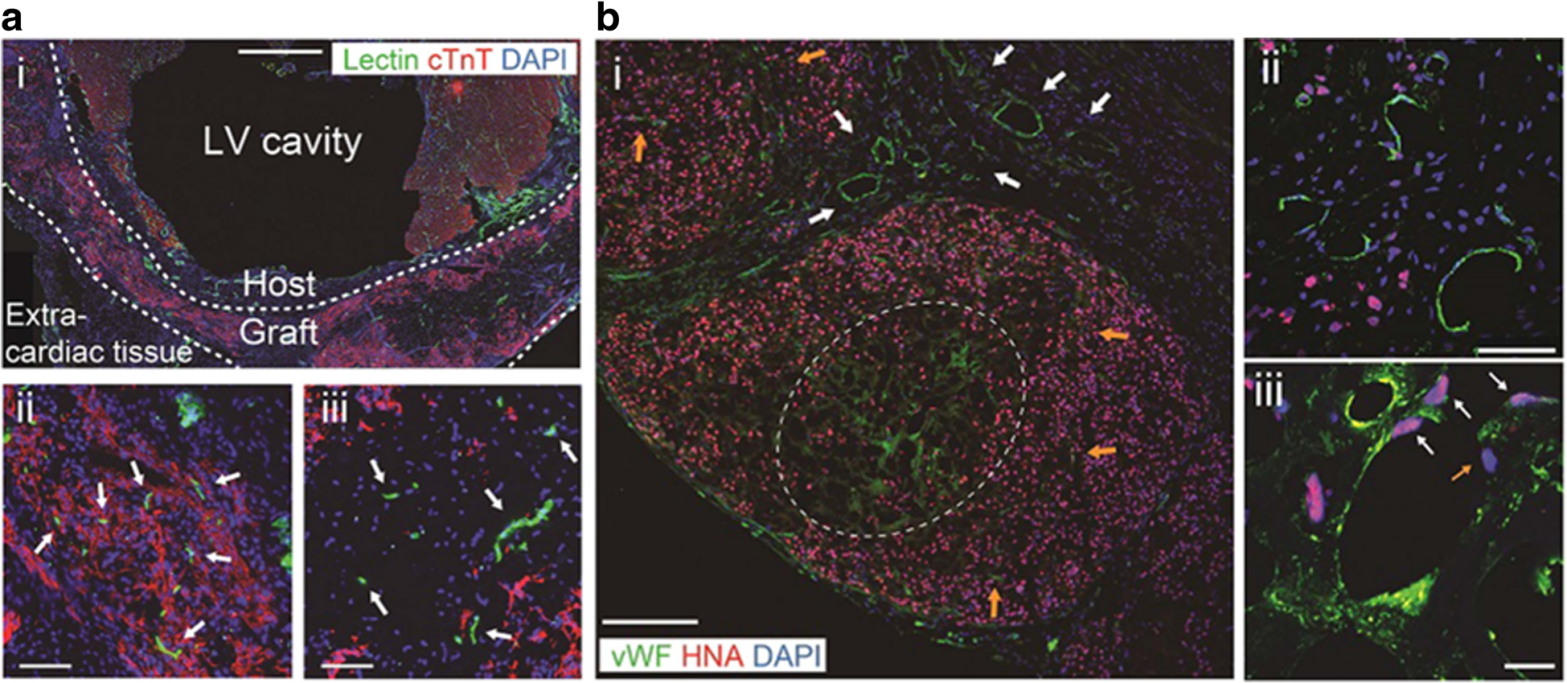
Vascular network formation after human iPS cell-derived engineered cardiac tissues (hiPSC-ECTs) transplantation onto a rat myocardial infarction model. **a** cTnT immunostaining for lectin (represents perfused vasculature)-perfused rat heart 4 weeks after implantation of hiPSC-ECTs. (i) Lower magnification image. (ii) and (iii) Higher magnification images. Perfused vasculature among (ii) and at central area (iii) of regenerated myocardium (arrows). **b** vWF (endothelial cell marker) and HNA double immunostaining. (i) Lower magnification image. White arrows indicate promoted capillary formation around grafted tissue. Orange arrows indicate penetrating vasculature. White dotted line indicates vasculature in the center of grafted tissue. (ii) and (iii) Higher magnification images. (ii) Prominent host-derived (HNA^−^) vascular formation around regenerated myocardium. (iii) chimeric vasculature composed of both host (HNA^−^; orange arrow) and graft (HNA^+^; white arrows) vascular cells. cTnT, cardiac troponin-T; DAPI, 4, 6 diamidino-2-phenylindole; LV, left ventricle; vWF, von Willebrand factor; HNA, human nucleic antigen. Scale bars 1 mm in (**a**) (i), 200 μm in (**b**) (i), 100 μm in (**a**) (ii, iii), 50 μm in (**b**) (ii), 20 μm in (**b**) (iii). Referred from reference no. 5 with modifications

The transplantation of hiPSC-ECTs onto a rat MI model revealed an increase of capillaries around the grafted tissue and a fair vascular network formation throughout the regenerated myocardium at 4 weeks after transplantation [5]. The injection of fluorescent dye-conjugated lectin via venous system of the host rat validated that the vascular network was functional to perfuse the regenerated myocardium (Fig. 2a). The immunohistochemical analyses for human nucleic antigen, specific for human cells, and von Willebrand factor, a specific marker for endothelial cells, showed that the vasculature inside the regenerated myocardium was composed of both host human and recipient rat cells (Fig. 2b). These results indicate that the transplanted hiPSC-ECTs can survive through the mechanism of vascular network formation between host and graft.

### Mechanisms and significance of perfusion among engineered 3D constructs in vivo

**Fig. 3:**
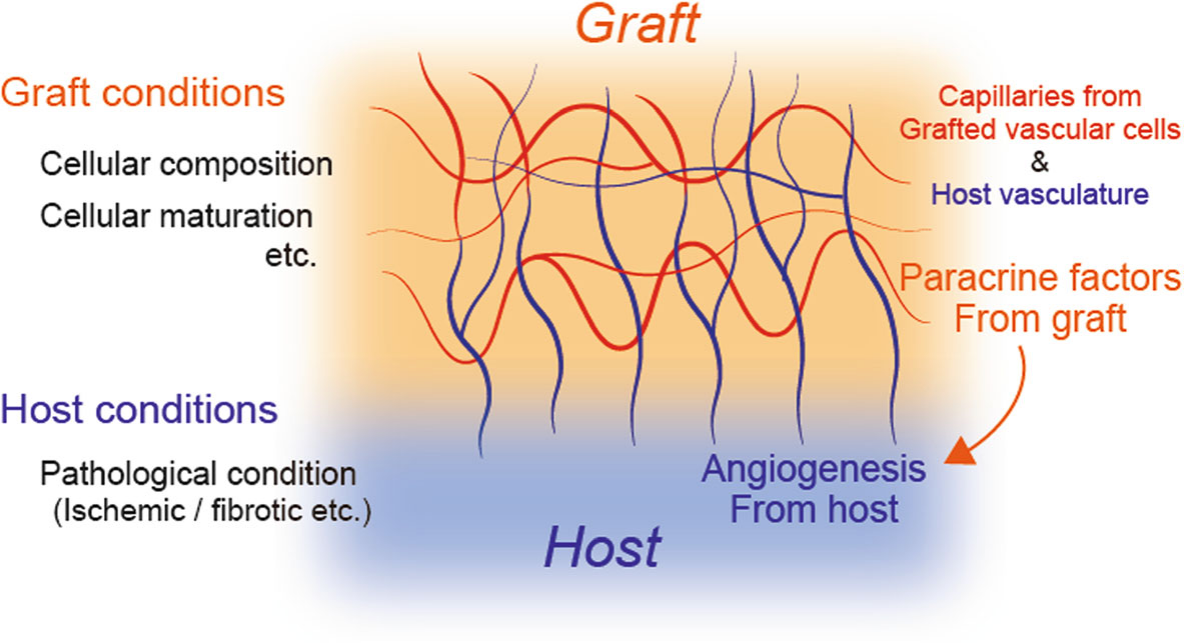
Schematic summary of the mechanism of capillary formation in 3D cardiac tissue. Biological interaction between host and graft results in capillary formation throughout the graft securing long-term graft survival

In 3D cardiac tissue formats introduced above, the microcapillary network formation throughout the tissue contributed to the long-term survival of the tissues. It is possible that the mechanism of vascular network formation in vivo includes two different biological processes: (1) Angiogenesis mediated by paracrine factors from transplanted engineered tissues, and (2) physical contribution of transplanted vascular cells which can be incorporated into newly formed vasculatures inside the regenerated myocardium. The sufficient vascular formation by the collaboration of these processes might be advantageous for long-term 3D graft survival and a successful cardiac regenerative therapy. On the other hand, the required extent of in vitro vascular formulation prior to in vivo transplantation is still controversial. It may depend on the balance of the in vitro conditions such as cellular composition/maturation or culture microenvironment, and in vivo conditions of transplanted site such as oxygen concentration or vascular supply which depends on disease conditions of the recipient heart (Fig. 3). It requires further investigations to optimize the suitable formulation for an efficient vascular network formation in vivo and functional integration between host and graft.

## Conclusion

In the present review, we introduced various strategies to induce microcapillaries for 3D engineered cardiac tissues aiming to promote the effectiveness of stem cell-based cardiac regenerative therapy. Further investigations for the post-transplantation vascularization are anticipated.
